# Enhancing Timing and Referral Pathways for Laparoscopic Cholecystectomy: A Two-Cycle Audit

**DOI:** 10.7759/cureus.98090

**Published:** 2025-11-29

**Authors:** Aaditi Hardenia, Hussein Ahmed, Alaa Hassan, Khaild Eltayeb, Ahmed ElKhadrawy

**Affiliations:** 1 General Surgery, Newham University Hospital, Barts Health NHS Trust, London, GBR

**Keywords:** acute cholecystitis, gallstone disease, laparoscopic cholecystectomy, quality improvement, referral pathway, surgical audit

## Abstract

Background: Laparoscopic cholecystectomy is the definitive treatment for symptomatic gallstone disease. National guidelines recommend surgery within two to six weeks following presentation to reduce complications and unplanned readmissions. This audit aimed to evaluate adherence to local guidance on timing and referral for laparoscopic cholecystectomy and assess the impact of service improvements. Methods: A two-cycle audit was conducted. The first cycle (July-August 2024, n=70) assessed the referral process, pre-assessment clinic (PAC) attendance, and booking timelines. Interventions included a structured cholecystectomy pathway, teaching sessions, and a standardised booking email. The second cycle (July-August 2025, n=50) reviewed outcomes following implementation. Key measures were PAC attendance, surgical booking timeliness, avoidance of unnecessary follow-up, and readmission rates. Results: In the first cycle, only 3% had PAC arranged, with a 36% readmission rate. In the second cycle, PAC improved to 16%, follow-ups were avoided in 30%, and readmissions were reduced to 22%. However, booking delays persisted, with 36% scheduled at three months and 40% not booked at all. Conclusion: Service interventions improved PAC attendance and reduced readmissions; however, delays in surgical booking remain the principal obstacle to achieving timely laparoscopic cholecystectomy. Further integration of the pathway into routine practice, along with addressing theatre capacity constraints, is essential to meet guideline standards and optimise patient outcomes.

## Introduction

Gallstone disease is one of the most prevalent gastrointestinal conditions, affecting approximately 10%-15% of adults in Western populations, and remains a leading cause of hospital admissions for biliary pathology [1,2]. Laparoscopic cholecystectomy is the gold standard treatment for symptomatic gallstone disease, offering faster recovery, shorter hospital stays, and lower complication rates compared to open surgery [3]. Timely surgical intervention plays a crucial role in reducing recurrent admissions, complications, and optimising the use of hospital resources.

National guidance from the National Institute for Health and Care Excellence (NICE CG188) recommends laparoscopic cholecystectomy within two weeks of initial presentation for acute cholecystitis and within six weeks for symptomatic gallstone disease to minimise recurrent attacks and unplanned readmissions [4]. The Royal College of Surgeons (RCS) and the World Society of Emergency Surgery (WSES) further emphasise the importance of early cholecystectomy, noting that delays can lead to biliary complications and repeated admissions [5,6].

Despite these recommendations, achieving timely surgery remains a challenge in many NHS trusts, with delays often attributed to limited theatre capacity, coordination inefficiencies, and patient pathway fragmentation [7]. Studies show that delayed laparoscopic cholecystectomy not only increases morbidity and healthcare costs but also impacts patient satisfaction and length of stay [8,9]. The objectives of this retrospective two-cycle audit were to quantify adherence to local and national standards for the timing and referral process of laparoscopic cholecystectomy, to assess compliance with the recommended two-to six-week surgical timeline, to evaluate pre-assessment clinic (PAC) attendance and booking efficiency, and to determine whether targeted pathway interventions improved service delivery and patient outcomes.

## Materials and methods

This was a retrospective two-cycle audit conducted at Newham University Hospital, Barts Health NHS Trust, London, UK. The aim was to assess compliance with local and national standards regarding the timing and referral process for laparoscopic cholecystectomy and to evaluate the impact of pathway interventions.

Study design and period

The study had two cycles. Cycle 1 was from July to August 2024 (n=70) and Cycle 2 from July to August 2025 (n=50).

This audit was conducted as a retrospective two-cycle quality-improvement project using a Plan-Do-Study-Act (PDSA) model. To improve clarity and reproducibility, the interventions introduced after Cycle 1 have been described in detail. The structured cholecystectomy pathway outlined required steps during initial assessment, referral, PAC scheduling, and theatre booking. Teaching sessions delivered to junior doctors, surgical coordinators, and administrative staff included pathway training, common sources of delay, and correct use of referral processes. A standardised booking email template was implemented, containing mandatory patient identifiers, clinical indication, results of investigations, and PAC requirements. Data were collected using the electronic patient record (EPR), theatre scheduling systems, and referral logs. Search filters included patient age ≥18 years, diagnosis of symptomatic gallstone disease, and referral for laparoscopic cholecystectomy. All eligible patients within each audit window were included. 

Inclusion criteria

All adult patients presenting with symptomatic gallstone disease (including biliary colic, cholecystitis, or gallstone pancreatitis) who were referred for laparoscopic cholecystectomy were included in the study.

Exclusion criteria

Patients undergoing elective cholecystectomy for reasons unrelated to gallstone disease or who had incomplete records were excluded.

Data collection

Patient data were obtained from EMRs, surgical lists, and referral logs. The following parameters were collected: date of initial presentation, referral and PAC attendance, time from referral to booking, whether a follow-up appointment was required to facilitate booking, and readmission occurrence within the same episode of care.

Standards

NICE CG188 [4] and local trust guidance recommend laparoscopic cholecystectomy within two weeks for acute cholecystitis and within six weeks for symptomatic gallstone disease.

Interventions implemented after Cycle 1

The interventions after Cycle 1 were as follows: 1. Development and dissemination of a standardised cholecystectomy pathway; 2. Teaching sessions for junior doctors, surgical coordinators, and booking staff; 3. Introduction of a structured referral and booking email template to improve communication and documentation.

Outcome measures

Primary outcomes were PAC attendance, time to surgical booking, and readmission rate. Secondary outcomes included the need for follow-up appointments and the proportion of patients booked within the recommended timeline.

Data analysis

Descriptive statistics were used to compare audit cycles. Categorical variables were presented as counts and percentages with corresponding sample sizes (n). Results were analysed and interpreted to assess the effectiveness of interventions.

All results are, therefore, reported as counts and percentages only, which is consistent with standard clinical audit methodology.

## Results

A total of 84 patients were included across both audit cycles, with 70 in Cycle 1 and 50 in Cycle 2. The mean age was similar between the two groups, and most patients presented with symptomatic gallstone disease or acute cholecystitis.

Cycle 1 findings (July-August 2024, n = 70)

**Figure 1:**
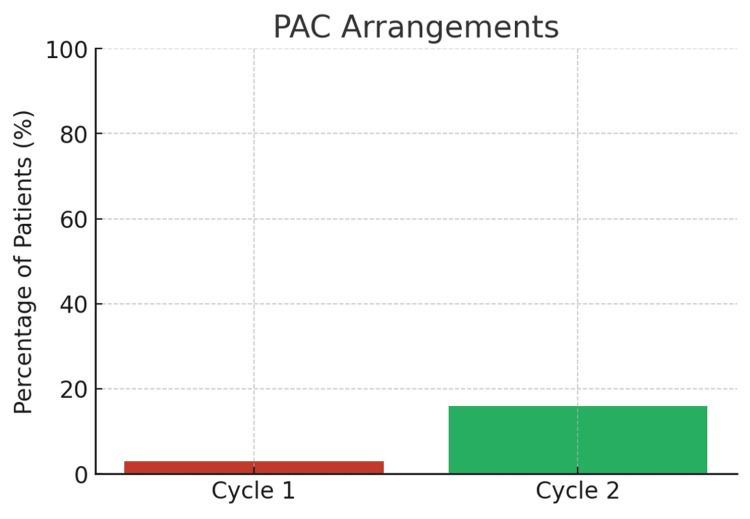
PAC arrangements across audit cycles PAC: pre-assessment clinic

**Figure 2:**
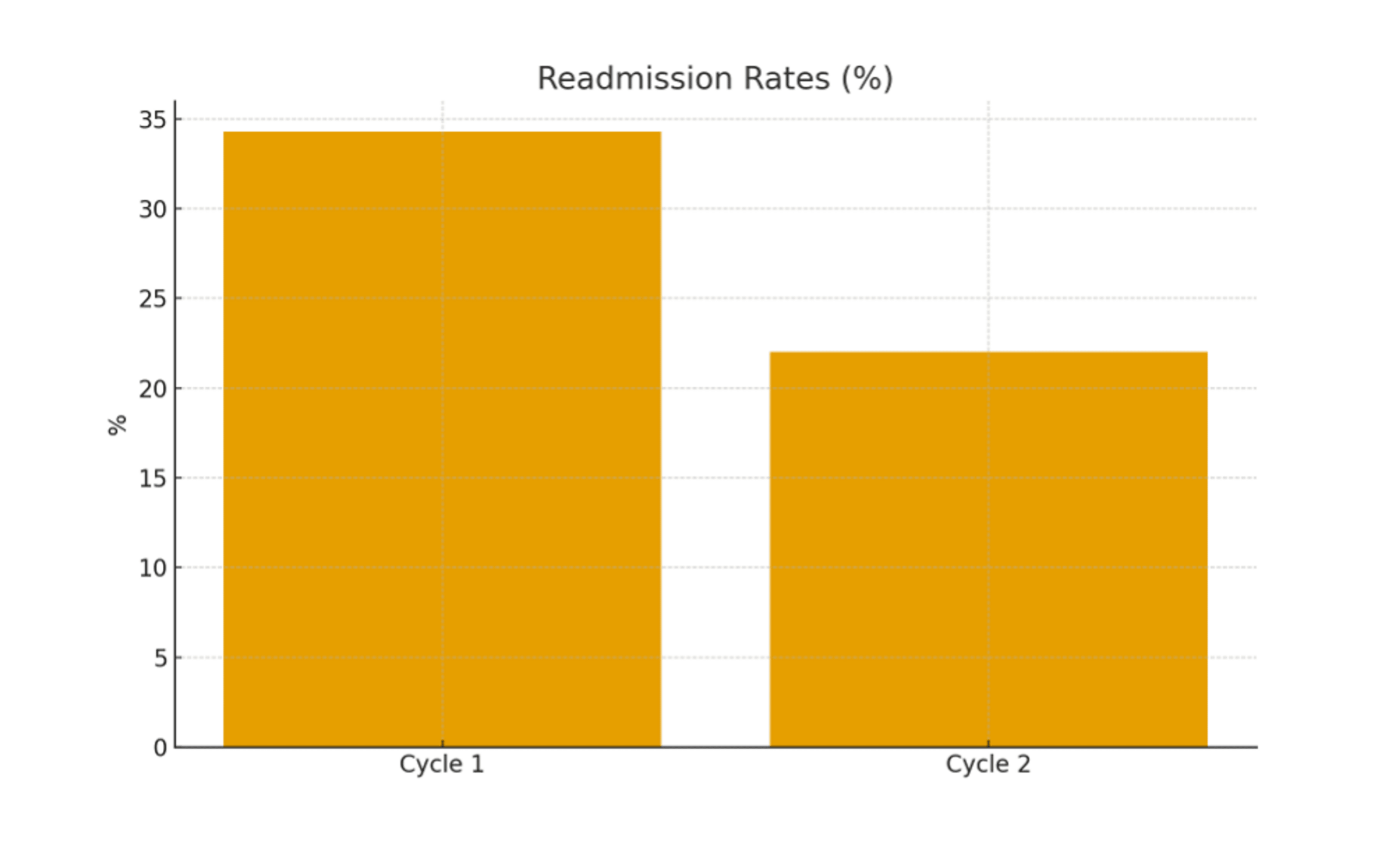
Comparison of readmission rates between cycles

Findings from Cycle 1, conducted between July and August 2024, revealed several inefficiencies within the referral and booking process. Only 3% (n = 2) of patients attended a PAC within the recommended two- to six-week period following presentation, while the remaining 97% (n = 68) required additional clinic visits or telephone consultations to complete booking and consent procedures. Timeliness of surgery was also below the expected standard, with a substantial proportion of patients experiencing delays that extended beyond the six-week national target. As a result, the readmission rate reached 36% (n = 25), predominantly due to recurrent biliary colic or cholecystitis during the waiting period. These findings highlighted the urgent need for a more structured referral pathway and enhanced multidisciplinary coordination to improve patient flow and reduce avoidable readmissions (Figures 1, 2).

Interventions introduced between cycles

Following the first cycle, three major interventions were introduced to optimise the cholecystectomy process. First, a standardised cholecystectomy pathway was established to provide clear guidance at every stage, from referral to surgery, ensuring a consistent and streamlined approach across the service. Second, targeted educational sessions were delivered to junior doctors, surgical coordinators, and administrative staff to promote uniform understanding and correct implementation of the pathway. Finally, a structured referral and booking email template was developed to improve the quality and clarity of communication between the clinical and booking teams, facilitating more efficient coordination and scheduling.

Cycle 2 findings (July-August 2025, n = 50)

The re-audit conducted after the implementation of the interventions demonstrated clear and measurable improvements across several domains. Attendance at the PAC increased to 16% (n = 8), compared with only 2.86% in the initial cycle, reflecting enhanced pathway adherence. Additionally, 30% (n = 15) of patients no longer required an extra follow-up appointment for consent or booking, highlighting greater efficiency in preoperative coordination.

**Figure 3:**
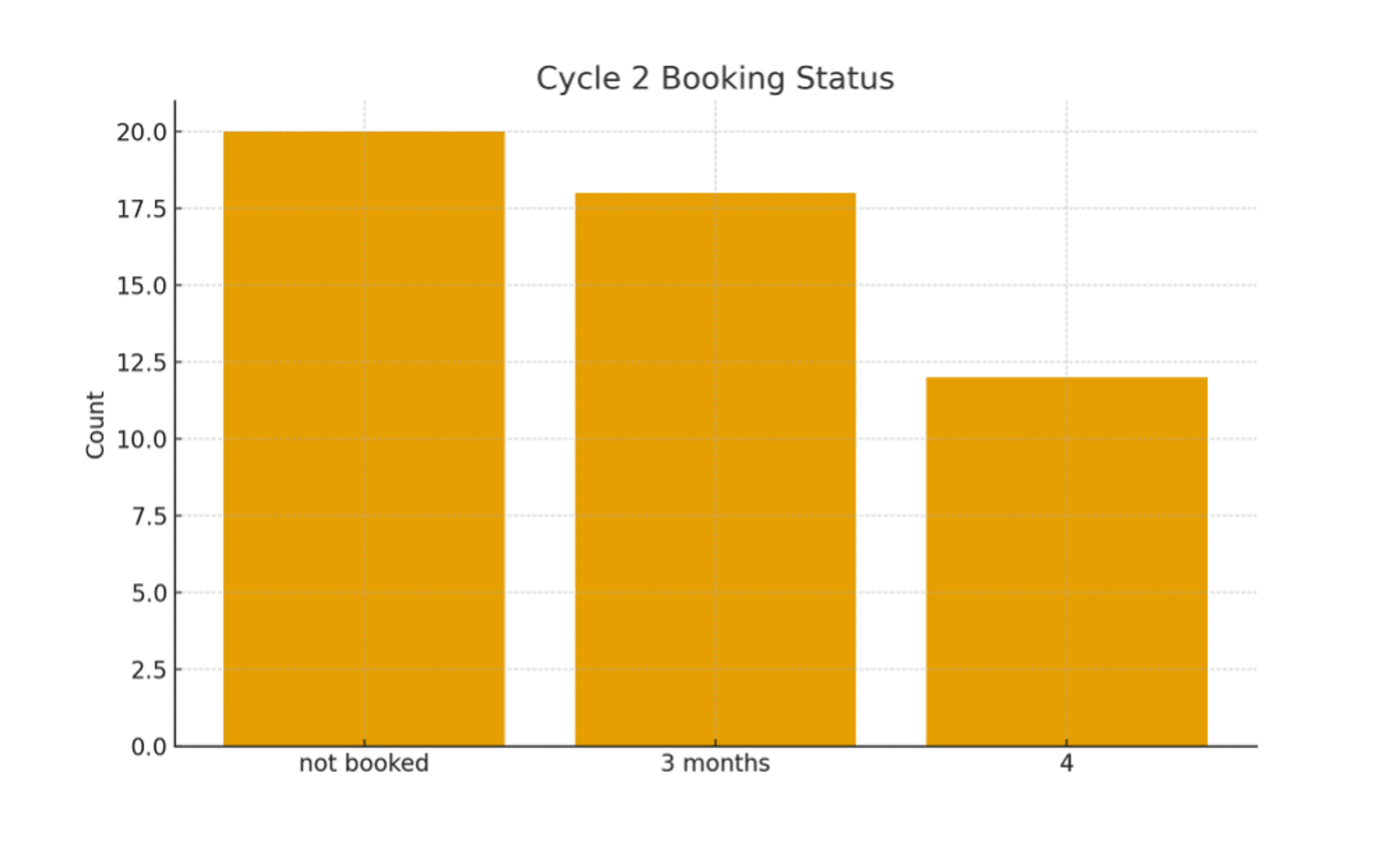
Booking status in Cycle 2

Although some booking delays remained, a proportion of patients experienced more timely scheduling, with 24% (n = 12) booked within two to four weeks and 36% (n = 18) scheduled at around three months. At the time of data collection, 40% (n = 20) were still awaiting theatre dates, as shown in Figure 3.

Notably, the readmission rate decreased to 22% (n = 11) in the second cycle, marking a 12-percentage-point reduction compared with the first cycle. Most readmissions occurred in patients whose operations had not yet been booked, indicating that earlier scheduling is likely to play a crucial role in reducing recurrent presentations and improving patient outcomes.

Comparative summary

Table 1 and Figures 1-3 collectively illustrate improvements in PAC utilisation and readmission reduction following implementation of the pathway interventions. Despite this progress, challenges remain in meeting national targets for surgery within six weeks, primarily due to persistent theatre capacity limitations.

**Table 1:** Comparative summary of key performance measures across audit cycles, demonstrating improvements in PAC attendance and readmission rates following implementation of pathway interventions. PAC: pre-assessment clinic

Parameter	Cycle 1 (n=70)	Cycle 2 (n=50)	Change
PAC attendance	2.86%	16%	Improved
Readmission rate	34.29%	22%	Improved
Not booked	—	20	Persistent issue
Booked at 3 months	—	18	Delayed
Booked at 4 weeks	—	12	Partial improvement

## Discussion

This audit demonstrates both the progress achieved and the ongoing challenges in optimising the timing and referral pathway for laparoscopic cholecystectomy at a district general hospital. The introduction of structured interventions, including a standardised pathway, a dedicated booking email template, and targeted education sessions, resulted in measurable improvements in pre-assessment attendance and a reduction in readmission rates. These findings support previous evidence that pathway standardisation and coordinated communication can improve surgical workflow and reduce avoidable delays [10].

According to national guidance from NICE (CG188) [4], patients with symptomatic gallstone disease or acute cholecystitis should ideally undergo laparoscopic cholecystectomy within two to six weeks of diagnosis to prevent recurrence and unplanned admissions. Achieving this target remains challenging across many NHS trusts due to limited theatre capacity, coordination inefficiencies, and competition for emergency operating slots, as also highlighted in the Royal College of Surgeons commissioning guidance [5] and the WSES guidelines on acute calculous cholecystitis [6]. Our findings closely mirror these national pressures: although administrative workflow improved, theatre booking delays persisted and continue to represent the primary bottleneck.

The reduction in readmission rates from 34.29% in Cycle 1 to 22% in Cycle 2 reflects the positive clinical impact of improved coordination. Evidence demonstrates that delayed laparoscopic cholecystectomy increases the risk of recurrent biliary colic, cholecystitis, and pancreatitis, resulting in higher healthcare utilisation [7]. Cochrane reviews have confirmed that early laparoscopic cholecystectomy reduces complications, length of stay, and readmissions without increasing conversion or adverse events [8], and further meta-analytic evidence supports early laparoscopic cholecystectomy as the optimal strategy for acute calculous cholecystitis [9].

Despite these improvements, a substantial proportion of patients in Cycle 2 (40%) had not yet been booked at the time of data collection, and 36% were scheduled at around three months, well beyond recommended timelines. These findings highlight systemic capacity limitations rather than clinical decision-making as the predominant barrier. National frameworks emphasise that laparoscopic cholecystectomy is a suitable day-case procedure, and both the British Association of Day Surgery (BADS) [11] and the Royal College of Surgeons Day Surgery Guidelines [12] advocate expanding day-case cholecystectomy to improve efficiency and maximise theatre throughput. Embedding laparoscopic cholecystectomy into day-surgery pathways, where clinically appropriate, could therefore reduce delays and enhance institutional capacity.

Although this audit was conducted at a single centre and results may not be fully generalisable, the trends demonstrated are consistent with national evidence and highlight areas where further improvement is both necessary and achievable. Future work should focus on increasing dedicated theatre access, leveraging day-case pathways, and expanding multidisciplinary collaboration to meet guideline standards and reduce biliary-related morbidity across the region.

## Conclusions

This two-cycle audit showed improvements in PAC utilisation and reductions in readmission following implementation of service changes. However, delays in surgical booking persist. Embedding the cholecystectomy pathway and checklist into routine care, alongside addressing theatre capacity, will be essential to achieving guideline standards and optimising patient outcomes.
